# Lobular Pneumonia

**Published:** 1846-09

**Authors:** 


*Lobular Pneumonia.*—One of the most important contributions, remarks Dr. West (*Report for* 1844-45), that has been made of late to our knowledge of infantile diseases, is the essay of MM. Bailly and Legendre, in the *Archives Gen. de. Med. Janv. Fevr. Mar.* 1844, on what is usually termed *lobular pneumonia.* They attack the generally received opinion of the inflammatory nature of this condition, which they regard as analogous to the state described by Jorg as atelectasis pulmonum. They conceive that this which they call the "*fœtal state*" of the lung is not invariably congenital, but that it may supervene afterwards, under certain circumstances.

The following conclusions embody the more important results of their researches:

1. In the bodies of children who have been rhachitic, weakly, or exhausted by previous disease, a number of the lobules of the lungs are found in a peculiar state of condensation, similar to that of the fœtal lung. 2. This fœtal state, which consists in occlusion of the vesicles, may result from the mere contractility of the tissue, or may depend on congestion of the vascular net work exterior to the vesicles. The former is the simple, the latter the congestive form of this affection. The congestive form is usually met with along the posterior border of the lungs, and generally accompanies catarrhal inflammation of the vesicles. 3. In either of these forms of the fœtal lung, insufflation reproduces, more or less completely, the natural condition of the lobules. 4. Though occasionally met with unassociated with inflammation, yet, in by far the majority of cases, this condition becomes developed under the influence of catarrh and catarrhal pneumonia. 5. When unattended with catarrh and involving only isolated lobules, this condition cannot be detected till after death, but in the new born infant it generally affects the lobar form, is attended by the physical signs of deficient respiration, and associated with the absence of all signs of constitutional reaction. 6. It is essentially different from hepatization, is produced by causes which interfere with the free performance of respiration, and is to be treated by remedies the reverse of antiphlogistic. 7. Lobular pneumonia, has, strictly speaking, no existence, since the action of inflammation is never confined to a single lobule, as is the case with the fœtal state of the lung. *Partial Pneumonia* would therefore be a fitter term. 8. Insufflation does not modify the patches of true hepatization, while the bronchi leading to such hepatized nodules are exempt from catarrh; two characters which distinguish partial pneumonia from the lobular engorgements of partial pneumonia. 9. True partial pneumonia is by no means common in children, though when hepatization does occur, in children under five years of age, it almost always affects the partial form. The statements, therefore, that have been made with reference to the variety of lobar pneumonia in infancy are correct; but almost all that has been said about the extreme frequency of lobular pneumonia at that age, must be taken as referring to the fœtal state of the lung. 10. Catarrhal pneumonia consists in the extension of the catarrhal inflammation from the bronchi to the pulmonary vesicles. This inflammation may affect healthy lobules, or those in the fœtal state. In the latter case it gives rise to appearances which have led to the supposition that these lobules were the seat of a parenchymatous inflammation. 11. Capillary bronchitis and generalized lobular pneumonia are but two forms of catarrhal pneumonia, which differ according as in the one the catarrhal element, or as in the other the lobular congestion predominates. 12. These facts explain why depletion was seldom appropriate to the treatment of what was called lobular pneumonia.

Simple, as the process was by which these results were obtained, no one had previously employed insufflation as a means of ascertaining the real nature of lobular pneumonia and carnification of the lung in children. Dr. West has repeated the experiments of MM. Bailly and Legendre on many occasions, and fully substantiates the correctness of their statements.

It is asserted by M. Bouchut, that even true hepatization may be removed by insufflation; in this, however, he is decidedly wrong. The hepatized portion may sometimes be made to assume a brighter color, but not to resume the texture of healthy lung, as is the case with lung in the fœtal state.—*Trans. of Col. of Physicians of Phil.*

